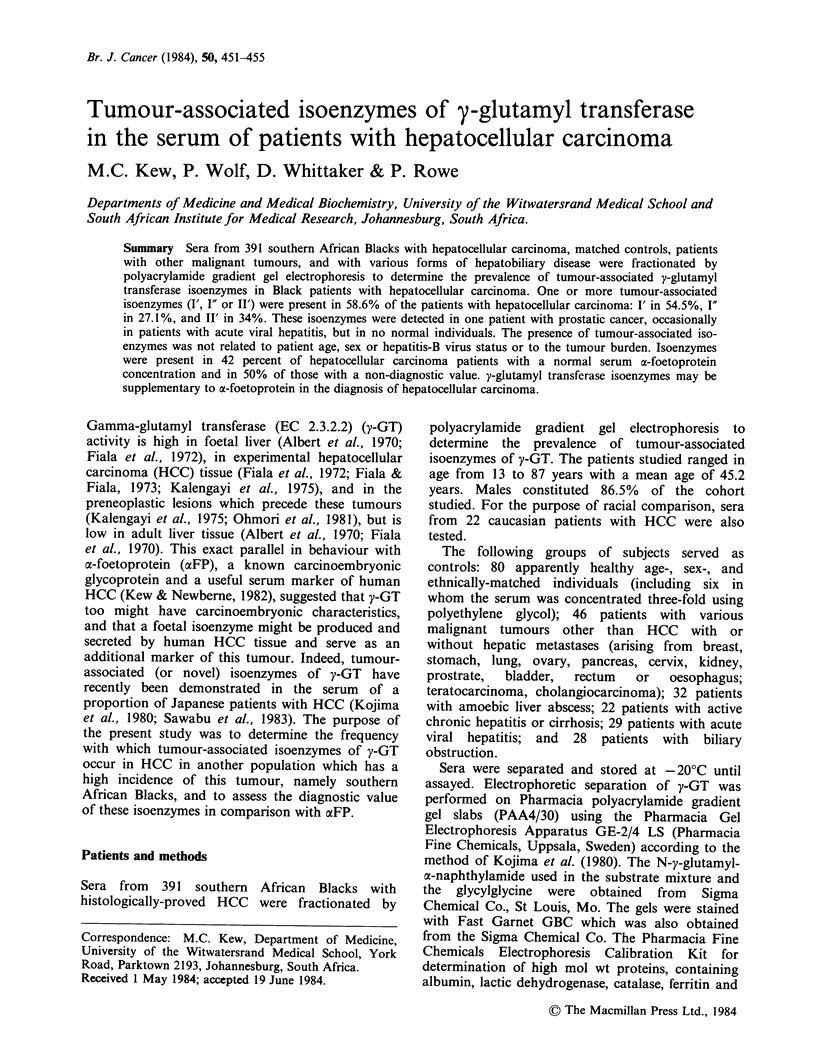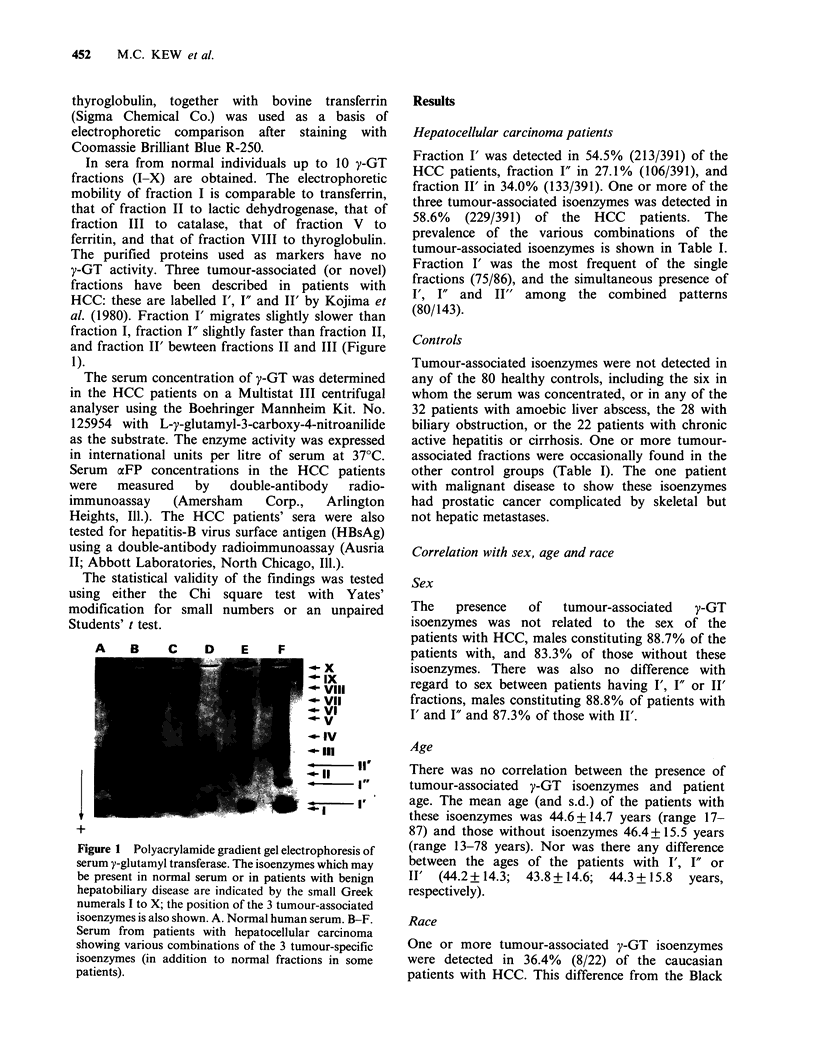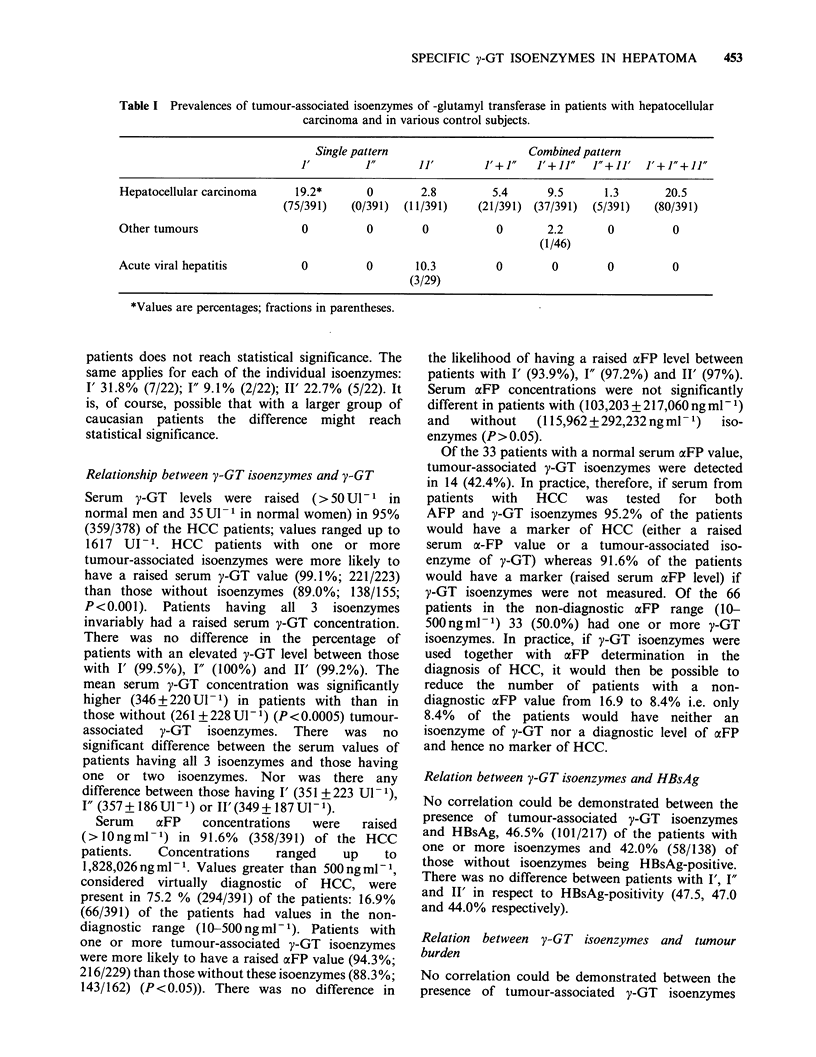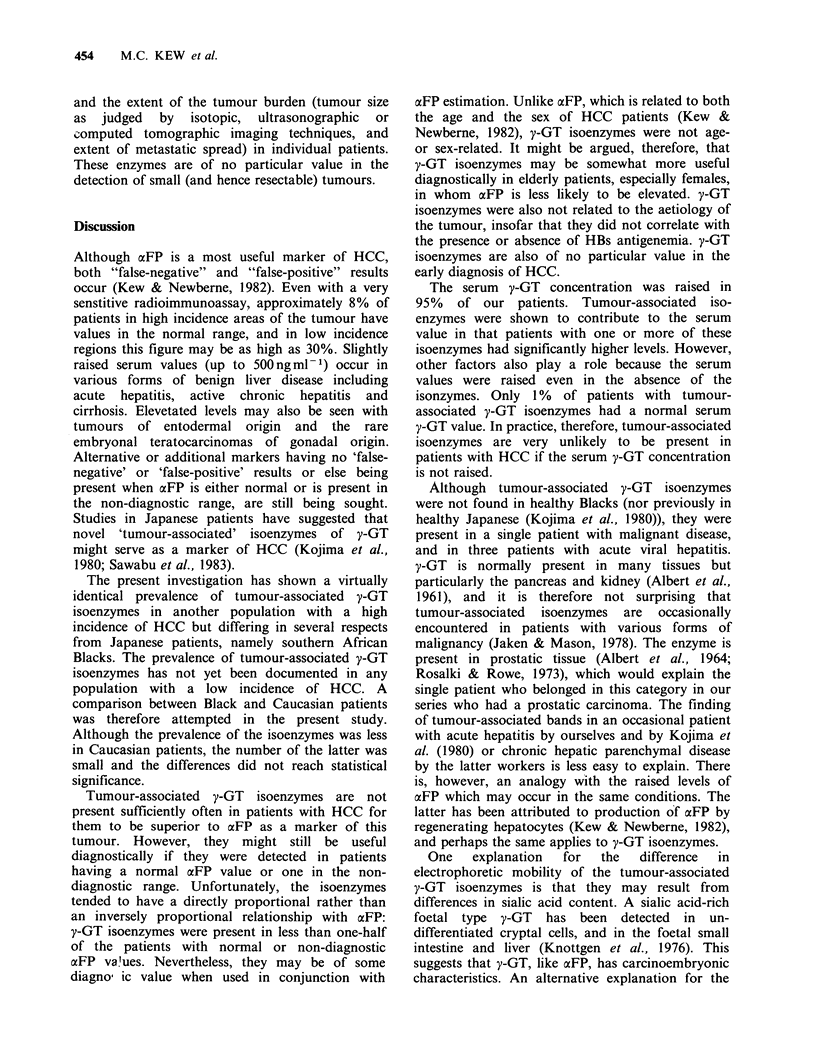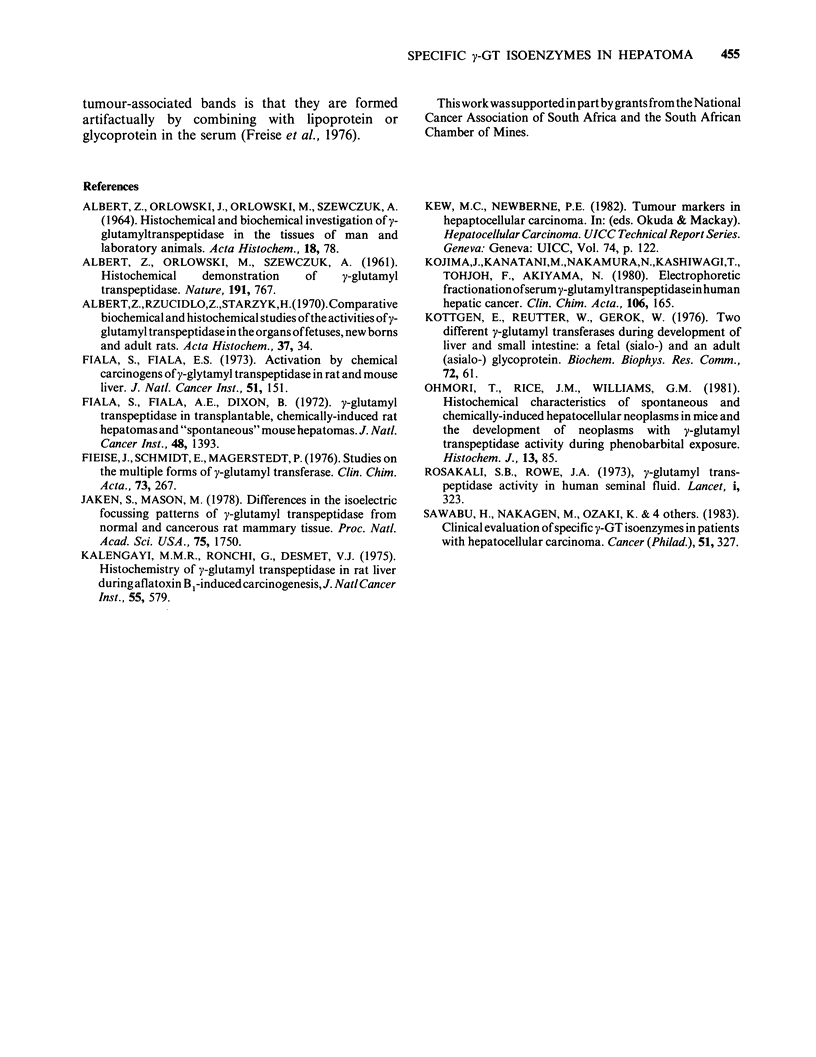# Tumour-associated isoenzymes of gamma-glutamyl transferase in the serum of patients with hepatocellular carcinoma.

**DOI:** 10.1038/bjc.1984.200

**Published:** 1984-10

**Authors:** M. C. Kew, P. Wolf, D. Whittaker, P. Rowe

## Abstract

**Images:**


Br. J. Cancer (1984), 50, 451–455

### Tumour-associated isoenzymes of γ-glutamyl transferase in the serum of patients with hepatocellular carcinoma

M.C. Kew, P. Wolf, D. Whittaker & P. Rowe

*Departments of Medicine and Medical Biochemistry, University of the Witwatersrand Medical School and South African Institute for Medical Research, Johannesburg, South Africa.*

**Summary** Sera from 391 southern African Blacks with hepatocellular carcinoma, matched controls, patients with other malignant tumours, and with various forms of hepatobiliary disease were fractionated by polyacrylamide gradient gel electrophoresis to determine the prevalence of tumour-associated γ-glutamyl transferase isoenzymes in Black patients with hepatocellular carcinoma. One or more tumour-associated isoenzymes (I′, I″ or II′) were present in 58.6% of the patients with hepatocellular carcinoma: I′ in 54.5%, I″ in 27.1%, and II′ in 34%. These isoenzymes were detected in one patient with prostatic cancer, occasionally in patients with acute viral hepatitis, but in no normal individuals. The presence of tumour-associated isoenzymes was not related to patient age, sex or hepatitis-B virus status or to the tumour burden. Isoenzymes were present in 42 percent of hepatocellular carcinoma patients with a normal serum α-foetoprotein concentration and in 50% of those with a non-diagnostic value. γ-glutamyl transferase isoenzymes may be supplementary to α-foetoprotein in the diagnosis of hepatocellular carcinoma.

Gamma-glutamyl transferase (EC 2.3.2.2) (γ-GT) activity is high in foetal liver (Albert *et al.*, 1970; Fiala *et al.*, 1972), in experimental hepatocellular carcinoma (HCC) tissue (Fiala *et al.*, 1972; Fiala & Fiala, 1973; Kalengayi *et al.*, 1975), and in the preneoplastic lesions which precede these tumours (Kalengayi *et al.*, 1975; Ohmori *et al.*, 1981), but is low in adult liver tissue (Albert *et al.*, 1970; Fiala *et al.*, 1970). This exact parallel in behaviour with α-foetoprotein (αFP), a known carcinoembryonic glycoprotein and a useful serum marker of human HCC (Kew & Newberne, 1982), suggested that γ-GT too might have carcinoembryonic characteristics, and that a foetal isoenzyme might be produced and secreted by human HCC tissue and serve as an additional marker of this tumour. Indeed, tumour-associated (or novel) isoenzymes of γ-GT have recently been demonstrated in the serum of a proportion of Japanese patients with HCC (Kojima *et al.*, 1980; Sawabu *et al.*, 1983). The purpose of the present study was to determine the frequency with which tumour-associated isoenzymes of γ-GT occur in HCC in another population which has a high incidence of this tumour, namely southern African Blacks, and to assess the diagnostic value of these isoenzymes in comparison with αFP.

### Patients and methods

Sera from 391 southern African Blacks with histologically-proved HCC were fractionated by polyacrylamide gradient gel electrophoresis to determine the prevalence of tumour-associated isoenzymes of γ-GT. The patients studied ranged in age from 13 to 87 years with a mean age of 45.2 years. Males constituted 86.5% of the cohort studied. For the purpose of racial comparison, sera from 22 caucasian patients with HCC were also tested.

The following groups of subjects served as controls: 80 apparently healthy age-, sex-, and ethnically-matched individuals (including six in whom the serum was concentrated three-fold using polyethylene glycol); 46 patients with various malignant tumours other than HCC with or without hepatic metastases (arising from breast, stomach, lung, ovary, pancreas, cervix, kidney, prostrate, bladder, rectum or oesophagus; teratocarcinoma, cholangiocarcinoma); 32 patients with amoebic liver abscess; 22 patients with active chronic hepatitis or cirrhosis; 29 patients with acute viral hepatitis; and 28 patients with biliary obstruction.

Sera were separated and stored at −20°C until assayed. Electrophoretic separation of γ-GT was performed on Pharmacia polyacrylamide gradient gel slabs (PAA4/30) using the Pharmacia Gel Electrophoresis Apparatus GE-2/4 LS (Pharmacia Fine Chemicals, Uppsala, Sweden) according to the method of Kojima *et al.* (1980). The N-γ-glutamyl-α-naphthylamide used in the substrate mixture and the glycylglycine were obtained from Sigma Chemical Co., St Louis, Mo. The gels were stained with Fast Garnet GBC which was also obtained from the Sigma Chemical Co. The Pharmacia Fine Chemicals Electrophoresis Calibration Kit for determination of high mol wt proteins, containing albumin, lactic dehydrogenase, catalase, ferritin and

Correspondence: M.C. Kew, Department of Medicine, University of the Witwatersrand Medical School, York Road, Parktown 2193, Johannesburg, South Africa.
Received 1 May 1984; accepted 19 June 1984.

© The Macmillan Press Ltd., 1984

thyroglobulin, together with bovine transferrin (Sigma Chemical Co.) was used as a basis of electrophoretic comparison after staining with Coomassie Brilliant Blue R-250.

In sera from normal individuals up to 10 γ-GT fractions (I–X) are obtained. The electrophoretic mobility of fraction I is comparable to transferrin, that of fraction II to lactic dehydrogenase, that of fraction III to catalase, that of fraction V to ferritin, and that of fraction VIII to thyroglobulin. The purified proteins used as markers have no γ-GT activity. Three tumour-associated (or novel) fractions have been described in patients with HCC: these are labelled I′, I″ and II′ by Kojima *et al.* (1980). Fraction I′ migrates slightly slower than fraction I, fraction I″ slightly faster than fraction II, and fraction II′ bewteen fractions II and III (Figure 1).

The serum concentration of γ-GT was determined in the HCC patients on a Multistat III centrifugal analyser using the Boehringer Mannheim Kit. No. 125954 with L-γ-glutamyl-3-carboxy-4-nitroanilide as the substrate. The enzyme activity was expressed in international units per litre of serum at 37°C. Serum αFP concentrations in the HCC patients were measured by double-antibody radioimmunoassay (Amersham Corp., Arlington Heights, Ill.). The HCC patients' sera were also tested for hepatitis-B virus surface antigen (HBsAg) using a double-antibody radioimmunoassay (Ausria II; Abbott Laboratories, North Chicago, Ill.).

The statistical validity of the findings was tested using either the Chi square test with Yates' modification for small numbers or an unpaired Students' *t* test.

**Figure 1** Polyacrylamide gradient gel electrophoresis of serum γ-glutamyl transferase. The isoenzymes which may be present in normal serum or in patients with benign hepatobiliary disease are indicated by the small Greek numerals I to X; the position of the 3 tumour-associated isoenzymes is also shown. A. Normal human serum. B–F. Serum from patients with hepatocellular carcinoma showing various combinations of the 3 tumour-specific isoenzymes (in addition to normal fractions in some patients).

### Results

### *Hepatocellular carcinoma patients*

Fraction I′ was detected in 54.5% (213/391) of the HCC patients, fraction I″ in 27.1% (106/391), and fraction II′ in 34.0% (133/391). One or more of the three tumour-associated isoenzymes was detected in 58.6% (229/391) of the HCC patients. The prevalence of the various combinations of the tumour-associated isoenzymes is shown in Table I. Fraction I′ was the most frequent of the single fractions (75/86), and the simultaneous presence of I′, I″ and II″ among the combined patterns (80/143).

### *Controls*

Tumour-associated isoenzymes were not detected in any of the 80 healthy controls, including the six in whom the serum was concentrated, or in any of the 32 patients with amoebic liver abscess, the 28 with biliary obstruction, or the 22 patients with chronic active hepatitis or cirrhosis. One or more tumour-associated fractions were occasionally found in the other control groups (Table I). The one patient with malignant disease to show these isoenzymes had prostatic cancer complicated by skeletal but not hepatic metastases.

### *Correlation with sex, age and race*

#### *Sex*

The presence of tumour-associated γ-GT isoenzymes was not related to the sex of the patients with HCC, males constituting 88.7% of the patients with, and 83.3% of those without these isoenzymes. There was also no difference with regard to sex between patients having I′, I″ or II′ fractions, males constituting 88.8% of patients with I′ and I″ and 87.3% of those with II′.

#### *Age*

There was no correlation between the presence of tumour-associated γ-GT isoenzymes and patient age. The mean age (and s.d.) of the patients with these isoenzymes was 44.6±14.7 years (range 17–87) and those without isoenzymes 46.4±15.5 years (range 13–78 years). Nor was there any difference between the ages of the patients with I′, I″ or II′ (44.2±14.3; 43.8±14.6; 44.3±15.8 years, respectively).

#### *Race*

One or more tumour-associated γ-GT isoenzymes were detected in 36.4% (8/22) of the caucasian patients with HCC. This difference from the Black

**Table I** Prevalences of tumour-associated isoenzymes of -glutamyl transferase in patients with hepatocellular carcinoma and in various control subjects.

| | *Single pattern* | | | *Combined pattern* | | | |
|---|---|---|---|---|---|---|---|
| | *I′* | *I″* | *II′* | *I′+I″* | *I′+II″* | *I″+II′* | *I′+I″+II″* |
| Hepatocellular carcinoma | 19.2* (75/391) | 0 (0/391) | 2.8 (11/391) | 5.4 (21/391) | 9.5 (37/391) | 1.3 (5/391) | 20.5 (80/391) |
| Other tumours | 0 | 0 | 0 | 0 | 2.2 (1/46) | 0 | 0 |
| Acute viral hepatitis | 0 | 0 | 10.3 (3/29) | 0 | 0 | 0 | 0 |

*Values are percentages; fractions in parentheses.

patients does not reach statistical significance. The same applies for each of the individual isoenzymes: I′ 31.8% (7/22); I″ 9.1% (2/22); II′ 22.7% (5/22). It is, of course, possible that with a larger group of caucasian patients the difference might reach statistical significance.

*Relationship between γ-GT isoenzymes and γ-GT*

Serum γ-GT levels were raised (>50 $\mathrm{Ul^{-1}}$ in normal men and 35 $\mathrm{Ul^{-1}}$ in normal women) in 95% (359/378) of the HCC patients; values ranged up to 1617 $\mathrm{UI^{-1}}$. HCC patients with one or more tumour-associated isoenzymes were more likely to have a raised serum γ-GT value (99.1%; 221/223) than those without isoenzymes (89.0%; 138/155; $P<0.001$). Patients having all 3 isoenzymes invariably had a raised serum γ-GT concentration. There was no difference in the percentage of patients with an elevated γ-GT level between those with I′ (99.5%), I″ (100%) and II′ (99.2%). The mean serum γ-GT concentration was significantly higher ($346 \pm 220\,\mathrm{Ul^{-1}}$) in patients with than in those without ($261 \pm 228\,\mathrm{Ul^{-1}}$) ($P<0.0005$) tumour-associated γ-GT isoenzymes. There was no significant difference between the serum values of patients having all 3 isoenzymes and those having one or two isoenzymes. Nor was there any difference between those having I′ ($351 \pm 223$ $\mathrm{Ul^{-1}}$), I″ ($357 \pm 186\,\mathrm{Ul^{-1}}$) or II′ ($349 \pm 187\,\mathrm{Ul^{-1}}$).

Serum αFP concentrations were raised (>10 $\mathrm{ng\,ml^{-1}}$) in 91.6% (358/391) of the HCC patients. Concentrations ranged up to 1,828,026 $\mathrm{ng\,ml^{-1}}$. Values greater than 500 $\mathrm{ng\,ml^{-1}}$, considered virtually diagnostic of HCC, were present in 75.2 % (294/391) of the patients: 16.9% (66/391) of the patients had values in the non-diagnostic range (10–500 $\mathrm{ng\,ml^{-1}}$). Patients with one or more tumour-associated γ-GT isoenzymes were more likely to have a raised αFP value (94.3%; 216/229) than those without these isoenzymes (88.3%; 143/162) ($P<0.05$)). There was no difference in the likelihood of having a raised αFP level between patients with I′ (93.9%), I″ (97.2%) and II′ (97%). Serum αFP concentrations were not significantly different in patients with ($103,203 \pm 217,060\,\mathrm{ng\,ml^{-1}}$) and without ($115,962 \pm 292,232\,\mathrm{ng\,ml^{-1}}$) isoenzymes ($P>0.05$).

Of the 33 patients with a normal serum αFP value, tumour-associated γ-GT isoenzymes were detected in 14 (42.4%). In practice, therefore, if serum from patients with HCC was tested for both AFP and γ-GT isoenzymes 95.2% of the patients would have a marker of HCC (either a raised serum α-FP value or a tumour-associated isoenzyme of γ-GT) whereas 91.6% of the patients would have a marker (raised serum αFP level) if γ-GT isoenzymes were not measured. Of the 66 patients in the non-diagnostic αFP range (10–500 $\mathrm{ng\,ml^{-1}}$) 33 (50.0%) had one or more γ-GT isoenzymes. In practice, if γ-GT isoenzymes were used together with αFP determination in the diagnosis of HCC, it would then be possible to reduce the number of patients with a non-diagnostic αFP value from 16.9 to 8.4% i.e. only 8.4% of the patients would have neither an isoenzyme of γ-GT nor a diagnostic level of αFP and hence no marker of HCC.

*Relation between γ-GT isoenzymes and HBsAg*

No correlation could be demonstrated between the presence of tumour-associated γ-GT isoenzymes and HBsAg, 46.5% (101/217) of the patients with one or more isoenzymes and 42.0% (58/138) of those without isoenzymes being HBsAg-positive. There was no difference between patients with I′, I″ and II′ in respect to HBsAg-positivity (47.5, 47.0 and 44.0% respectively).

*Relation between γ-GT isoenzymes and tumour burden*

No correlation could be demonstrated between the presence of tumour-associated γ-GT isoenzymes and the extent of the tumour burden (tumour size as judged by isotopic, ultrasonographic or computed tomographic imaging techniques, and extent of metastatic spread) in individual patients. These enzymes are of no particular value in the detection of small (and hence resectable) tumours.

### Discussion

Although αFP is a most useful marker of HCC, both "false-negative" and "false-positive" results occur (Kew & Newberne, 1982). Even with a very senstitive radioimmunoassay, approximately 8% of patients in high incidence areas of the tumour have values in the normal range, and in low incidence regions this figure may be as high as 30%. Slightly raised serum values (up to $500 \, ng \, ml^{-1}$) occur in various forms of benign liver disease including acute hepatitis, active chronic hepatitis and cirrhosis. Elevetated levels may also be seen with tumours of entodermal origin and the rare embryonal teratocarcinomas of gonadal origin. Alternative or additional markers having no 'false-negative' or 'false-positive' results or else being present when αFP is either normal or is present in the non-diagnostic range, are still being sought. Studies in Japanese patients have suggested that novel 'tumour-associated' isoenzymes of γ-GT might serve as a marker of HCC (Kojima *et al.*, 1980; Sawabu *et al.*, 1983).

The present investigation has shown a virtually identical prevalence of tumour-associated γ-GT isoenzymes in another population with a high incidence of HCC but differing in several respects from Japanese patients, namely southern African Blacks. The prevalence of tumour-associated γ-GT isoenzymes has not yet been documented in any population with a low incidence of HCC. A comparison between Black and Caucasian patients was therefore attempted in the present study. Although the prevalence of the isoenzymes was less in Caucasian patients, the number of the latter was small and the differences did not reach statistical significance.

Tumour-associated γ-GT isoenzymes are not present sufficiently often in patients with HCC for them to be superior to αFP as a marker of this tumour. However, they might still be useful diagnostically if they were detected in patients having a normal αFP value or one in the non-diagnostic range. Unfortunately, the isoenzymes tended to have a directly proportional rather than an inversely proportional relationship with αFP: γ-GT isoenzymes were present in less than one-half of the patients with normal or non-diagnostic αFP values. Nevertheless, they may be of some diagno[illegible]ic value when used in conjunction with αFP estimation. Unlike αFP, which is related to both the age and the sex of HCC patients (Kew & Newberne, 1982), γ-GT isoenzymes were not age- or sex-related. It might be argued, therefore, that γ-GT isoenzymes may be somewhat more useful diagnostically in elderly patients, especially females, in whom αFP is less likely to be elevated. γ-GT isoenzymes were also not related to the aetiology of the tumour, insofar that they did not correlate with the presence or absence of HBs antigenemia. γ-GT isoenzymes are also of no particular value in the early diagnosis of HCC.

The serum γ-GT concentration was raised in 95% of our patients. Tumour-associated isoenzymes were shown to contribute to the serum value in that patients with one or more of these isoenzymes had significantly higher levels. However, other factors also play a role because the serum values were raised even in the absence of the isonzymes. Only 1% of patients with tumour-associated γ-GT isoenzymes had a normal serum γ-GT value. In practice, therefore, tumour-associated isoenzymes are very unlikely to be present in patients with HCC if the serum γ-GT concentration is not raised.

Although tumour-associated γ-GT isoenzymes were not found in healthy Blacks (nor previously in healthy Japanese (Kojima *et al.*, 1980)), they were present in a single patient with malignant disease, and in three patients with acute viral hepatitis. γ-GT is normally present in many tissues but particularly the pancreas and kidney (Albert *et al.*, 1961), and it is therefore not surprising that tumour-associated isoenzymes are occasionally encountered in patients with various forms of malignancy (Jaken & Mason, 1978). The enzyme is present in prostatic tissue (Albert *et al.*, 1964; Rosalki & Rowe, 1973), which would explain the single patient who belonged in this category in our series who had a prostatic carcinoma. The finding of tumour-associated bands in an occasional patient with acute hepatitis by ourselves and by Kojima *et al.* (1980) or chronic hepatic parenchymal disease by the latter workers is less easy to explain. There is, however, an analogy with the raised levels of αFP which may occur in the same conditions. The latter has been attributed to production of αFP by regenerating hepatocytes (Kew & Newberne, 1982), and perhaps the same applies to γ-GT isoenzymes.

One explanation for the difference in electrophoretic mobility of the tumour-associated γ-GT isoenzymes is that they may result from differences in sialic acid content. A sialic acid-rich foetal type γ-GT has been detected in undifferentiated cryptal cells, and in the foetal small intestine and liver (Knottgen *et al.*, 1976). This suggests that γ-GT, like αFP, has carcinoembryonic characteristics. An alternative explanation for the tumour-associated bands is that they are formed artifactually by combining with lipoprotein or glycoprotein in the serum (Freise *et al.*, 1976).

This work was supported in part by grants from the National Cancer Association of South Africa and the South African Chamber of Mines.

**References**

ALBERT, Z., ORLOWSKI, J., ORLOWSKI, M., SZEWCZUK, A. (1964). Histochemical and biochemical investigation of γ-glutamyltranspeptidase in the tissues of man and laboratory animals. *Acta Histochem.*, **18**, 78.

ALBERT, Z., ORLOWSKI, M., SZEWCZUK, A. (1961). Histochemical demonstration of γ-glutamyl transpeptidase. *Nature*, **191**, 767.

ALBERT, Z., RZUCIDLO, Z., STARZYK, H. (1970). Comparative biochemical and histochemical studies of the activities of γ-glutamyl transpeptidase in the organs of fetuses, new borns and adult rats. *Acta Histochem.*, **37**, 34.

FIALA, S., FIALA, E.S. (1973). Activation by chemical carcinogens of γ-glytamyl transpeptidase in rat and mouse liver. *J. Natl. Cancer Inst.*, **51**, 151.

FIALA, S., FIALA, A.E., DIXON, B. (1972). γ-glutamyl transpeptidase in transplantable, chemically-induced rat hepatomas and "spontaneous" mouse hepatomas. *J. Natl. Cancer Inst.*, **48**, 1393.

FIEISE, J., SCHMIDT, E., MAGERSTEDT, P. (1976). Studies on the multiple forms of γ-glutamyl transferase. *Clin. Chim. Acta.*, **73**, 267.

JAKEN, S., MASON, M. (1978). Differences in the isoelectric focussing patterns of γ-glutamyl transpeptidase from normal and cancerous rat mammary tissue. *Proc. Natl. Acad. Sci. USA.*, **75**, 1750.

KALENGAYI, M.M.R., RONCHI, G., DESMET, V.J. (1975). Histochemistry of γ-glutamyl transpeptidase in rat liver during aflatoxin $B_1$-induced carcinogenesis, *J. Natl Cancer Inst.*, **55**, 579.

KEW, M.C., NEWBERNE, P.E. (1982). Tumour markers in hepaptocellular carcinoma. In: (eds. Okuda & Mackay). *Hepatocellular Carcinoma. UICC Technical Report Series. Geneva:* Geneva: UICC, Vol. 74, p. 122.

KOJIMA, J., KANATANI, M., NAKAMURA, N., KASHIWAGI, T., TOHJOH, F., AKIYAMA, N. (1980). Electrophoretic fractionation of serum γ-glutamyl transpeptidase in human hepatic cancer. *Clin. Chim. Acta.*, **106**, 165.

KOTTGEN, E., REUTTER, W., GEROK, W. (1976). Two different γ-glutamyl transferases during development of liver and small intestine: a fetal (sialo-) and an adult (asialo-) glycoprotein. *Biochem. Biophys. Res. Comm.*, **72**, 61.

OHMORI, T., RICE, J.M., WILLIAMS, G.M. (1981). Histochemical characteristics of spontaneous and chemically-induced hepatocellular neoplasms in mice and the development of neoplasms with γ-glutamyl transpeptidase activity during phenobarbital exposure. *Histochem. J.*, **13**, 85.

ROSAKALI, S.B., ROWE, J.A. (1973), γ-glutamyl transpeptidase activity in human seminal fluid. *Lancet*, **i**, 323.

SAWABU, H., NAKAGEN, M., OZAKI, K. & 4 others. (1983). Clinical evaluation of specific γ-GT isoenzymes in patients with hepatocellular carcinoma. *Cancer* (*Philad.*), **51**, 327.